# Intraoperative CT for Neuronavigation Guidance and Confirmation of Foramen Ovale Cannulation for Glycerol Trigeminal Rhizotomy: A Technical Report and Case Series

**DOI:** 10.7759/cureus.8100

**Published:** 2020-05-13

**Authors:** Meena Thatikunta, Jessica Eaton, Mohammed Nuru, Haring J Nauta

**Affiliations:** 1 Neurosurgery, University of Louisville Hospital, Louisville, USA; 2 Neurosurgery, University of Washington, Seattle, USA; 3 Neurosurgery, University of Louisville School of Medicine, Louisville, USA

**Keywords:** trigeminal neuralgia, intraoperative imaging, percutaneous approaches, glycerol rhizotomy, neuronavigation, stereotactic methods, facial pain, foramen ovale, meckel's cave

## Abstract

Glycerol rhizotomy was originally described as an initial surgical treatment for trigeminal neuralgia after the failure of medical therapy. Here we describe its use as a salvage procedure, typically after failure of multiple other modalities including microvascular decompression, stereotactic radiosurgery, and/or other percutaneous procedures. Foramen ovale cannulation as a “salvage procedure” may be complicated by lack of cerebrospinal fluid (CSF) return despite adequate cannulation of the foramen ovale, making conventional fluoroscopic confirmation of adequate needle placement less certain. In this article, we describe the application of intraoperative CT, fused with high-resolution preoperative CT/MRI for neuronavigation to accurately cannulate the foramen ovale and Meckel's cave for glycerol rhizotomy. Intraoperative CT, again fused with high-resolution preoperative CT and MRI studies, was then used to confirm accurate trajectory through the foramen ovale and the adequate location of the needle tip in Meckel's cave before injecting glycerol. We present our initial experience with 14 patients who underwent glycerol rhizotomy by these techniques depending on intraoperative CT. It appears that intraoperative CT-guided neuronavigation provides a practical, reliable, and accurate route to the foramen ovale and aids in the confirmation of adequate needle placement even when there is a lack of CSF return. These methods may be especially useful for difficult cannulations typical in salvage procedures. In an era of feasible intraoperative guidance, with advanced stereotactic planning software allowing the fusion of intraoperative CT with high-resolution preoperative CT and MRI datasets, these techniques can be applied to foramen ovale cannulation for glycerol rhizotomy without major modification.

## Introduction

Background

Glycerol rhizotomy presents the technical challenge of accurate cannulation through a fluoroscopic percutaneous approach. Fluoroscopic guidance of the skull base has its own associated learning curve. Even in experienced hands, cannulation of the foramen ovale using Hartel’s approach is associated with a technical failure rate of 1% to 5% with fluoroscopy [1]. Cannulation has been reported as unsuccessful in 2.7% to 8% of procedures [2,3]. With use of intraoperative CT, cannulation for glycerol rhizotomy may become a more attractive option for both neurosurgeons and patients.

Multiple groups have demonstrated the use of intraoperative neuronavigation with CT and/or MRI in radiofrequency ablation for trigeminal neuralgia, with one group reporting a statistically significant difference in the distance they advanced the needle using navigation versus fluoroscopy, implying more accurate localization and trajectory [4-6,7]. In 2014, Georgiopoulos et al. presented a technical report on five patients who underwent unsuccessful fluoroscopy-guided cannulation and subsequent successful CT-guided cannulation for balloon compression rhizotomy [2]. In 2017, Qiu et al. cannulated five cadaveric foramen ovale with the use of ultrasound and pre-procedure CT-fused imaging [1]. To the best of our knowledge, there are no technical reports on glycerol rhizotomy using intraoperative CT for both neuronavigation guidance and intraoperative confirmation of adequate needle placement.

The advantages of glycerol rhizotomy are well-described elsewhere. Briefly, glycerol rhizotomy is well-suited for elderly or medically frail patients who do not desire craniotomy for patients who cannot cooperate for intraoperative physiological testing (as needed for radiofrequency ablation). Furthermore, glycerol has a low incidence of post-operative corneal sensory loss or deafferentation pain and is easily repeated and tolerated in the same patient [8]. This is especially useful in cases with V1 involvement. In modern practice, glycerol rhizotomy has emerged as more of a "salvage" procedure for those who have failed other surgical treatments. Alternatively, the procedure may also be a reasonably attractive choice for patients with V1 pain who simply do not desire craniotomy despite vascular compression [3].

Foramen ovale cannulation is required for radiofrequency ablation, balloon compression, and glycerol rhizotomy. Safe and accurate cannulation is dependent on (1) surgeon experience, (2) normal and anatomical variants, and (3) radiographic guidance.

Surgeon experience

Modern-day cannulation most closely resembles Hartel's anterior percutaneous approach, in which the needle is advanced between the coronoid process of the mandible and the lateral pterygoid plate [9]. By this technique, the needle is typically inserted at a point 2.5 cm lateral to the corner of the mouth and 1 cm inferior to the plane parallel to the occlusal layer of the teeth. The needle should be angled toward the intersection of the vertical plane intersecting the ipsilateral pupil and the horizontal plane along the inferior zygomatic border, 3 cm anterior to the tragus. The needle should engage at a depth of 6 to 8 cm [10]. If malpositioning occurs, then the needle is likely too lateral or too medial.

Haptic feedback is an important component of any percutaneous technique and is particularly relevant in the case of foramen ovale cannulation. Confident and accurate localization is built through repetitive cannulation. Such practice may be gained through cadaveric laboratories; however, access to such experiences may be limited. Less experienced surgeons may encounter difficulty with cannulation for such reasons. Additionally, young neurosurgeons are increasingly trained with intraoperative neuronavigation and may be more confident with such visualization paired with haptic feedback.

Normal and anatomic variants

The size and shape of the foramen ovale changes throughout a human’s lifetime and may vary from patient to patient. The long axis of the foramen maximally measures 7.2 mm in adults, with a width of up to 3.7 mm [11]. Anatomical variations include a bony division into two to three compartments in 4.5% and a range of shapes from round to almond-shaped to slit-like [12,13]. Anatomical studies have suggested that other abnormalities, including ossification of the pterygospinous or pterygoalar ligaments, ragged foramina edges, and small bone spur, may be present [14]. Around 25.7% of foramen ovale may show developmental anomaly, including bony spine, tubercle, spur, or narrow shape, which may prohibit cannulation [12].

The Gasserian ganglion is located in Meckel’s cave at the apex of the petrous bone, medial to the cavernous sinus. The mean distance from the terminal end of the foramen ovale is 6 mm; however, there is significant variation in the length of the root of the trigeminal ganglion, as well as the anteroposterior position of the ganglion within the cave, affecting the depth the needle must reach in order to effectively perform the procedure [15].

Radiographic guidance 

Pollock and Potter explored fluoroscopy in 1916 and injected alcohol into cadavers. It was not until the 1930s that fluoroscopy was applied to patients for this purpose. Traditional radiographic guidance includes the use of live fluoroscopy and radiopaque spinal needle. Under lateral fluoroscopic guidance, the needle is adequately positioned when approximately 1 cm behind the posterior clinoid along the angle of the petrous bone. CT navigation allows not only allows easier cannulation of the foramen ovale but also avoidance of damage to the structures surrounding the trigeminal ganglion, which include the middle meningeal artery, the contents of the jugular foramen, and the carotid artery [1]. The most serious potential complication is damage to the internal carotid artery. Return of cerebrospinal fluid (CSF) is typically an indication that the needle is within Meckel's cave; however, CSF flow can also occur when the needle is positioned too lateral and passes through the middle fossa floor, landing in the subtemporal or subdural space [16]. This may be more likely in patients in whom the middle fossa floor is especially thin or osteoporotic. Exceptionally, the inferior orbital fissure and the jugular foramen may also be damaged during attempted cannulation [17,18].

The current standard technique uses fluoroscopy to obtain anteroposterior imaging of the foramen ovale and a lateral projection to study the trajectory depth [16]. While effective in localization, live fluoroscopy causes radiation exposure to the patient, surgeon, and operating staff. From an anatomical perspective, fluoroscopy provides guidance only in two planes and at non-simultaneous times (unless performed in a biplane suite). This drawback magnifies for patients with variant anatomy, as described previously, and may preclude cannulation entirely in a small subset of patients. With the advent of intraoperative CT neuronavigation, such obstacles may be anticipated and overcome.

## Technical report

Methods 

Institutional Review Board approval was obtained from the University of Louisville. A retrospective review of cases from January 2017 to January 2019 for glycerol rhizotomy cases revealed 14 cases. One patient was lost to follow-up post-operatively and was excluded from any follow-up analyses. All cases were reviewed for the following characteristics: age, gender, TN-1 versus TN-2 symptoms, current medical treatments, previous surgical treatments, ability to engage the foramen, presence of CSF return intraoperatively, pain outcomes at follow-up, and time interval to repeat procedure in cases of delayed pain recurrence. Time to recurrence was not well characterized due to the data quality encountered retrospectively; time to repeat operation was used as a surrogate due to its objective nature. Immediate post-operative pain relief was not routinely documented in the medical record and therefore this was also not analyzed; however, we note that in patients who report pain relief, it was typically experienced immediately in the recovery bay. In cases of anticipated or encountered technical difficulty, surgical course and images were reviewed with the surgeon of record for the understanding of the factors involved.

Technique 

Preoperatively, patients undergo a high-resolution thin-slice stereotactic protocol head CT and T2-weighted MRI. These studies are very helpful because the resolution of a CT scanner in the operating room (Airo®, Brainlab AG, Munich, Germany, or O-ArmTM, Medtronic, Minneapolis, MN, USA) is less than ideal for identifying an atypical foramen ovale or the boundaries of Meckel's cave. Patients who have previously undergone stereotactic radiosurgery may already have the necessary high-resolution preoperative studies available. At surgery, the patient is placed under general anesthesia with a laryngeal mask airway (LMA) or endotracheal airway. The patient is positioned supine in a radiolucent pin fixation device. The Airo® System was used in this series for intraoperative CT performed at the highest resolution setting with a reference array attached to the head holder. This intraoperative scan was then fused with preoperative CT and MRI. The foramen ovale is typically best identified on the fused preoperative CT and is then set as the entry point. The target point is chosen in Meckel's cave to establish the trajectory. The operative side of the face is then prepped and draped in a sterile fashion. A Brainlab tracking array is then attached to the hub of an 18-gauge spinal needle and registered as a trackable object to the neuronavigation station. Accurate neuronavigation is confirmed with facial landmarks.

**Figure 1:**
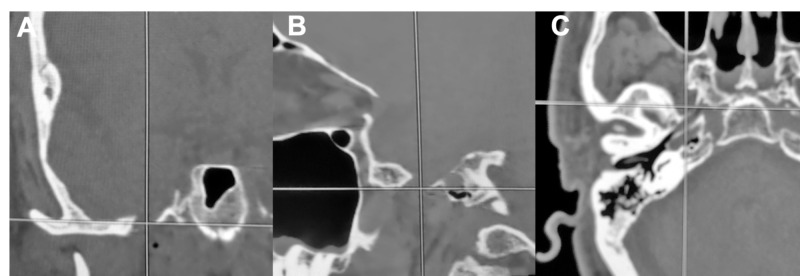
Coronal (A), Sagittal (B), and Axial (C) CT images with superimposed cross-hair marking the intended target for needle entry into the foramen ovale.

**Figure 2:**
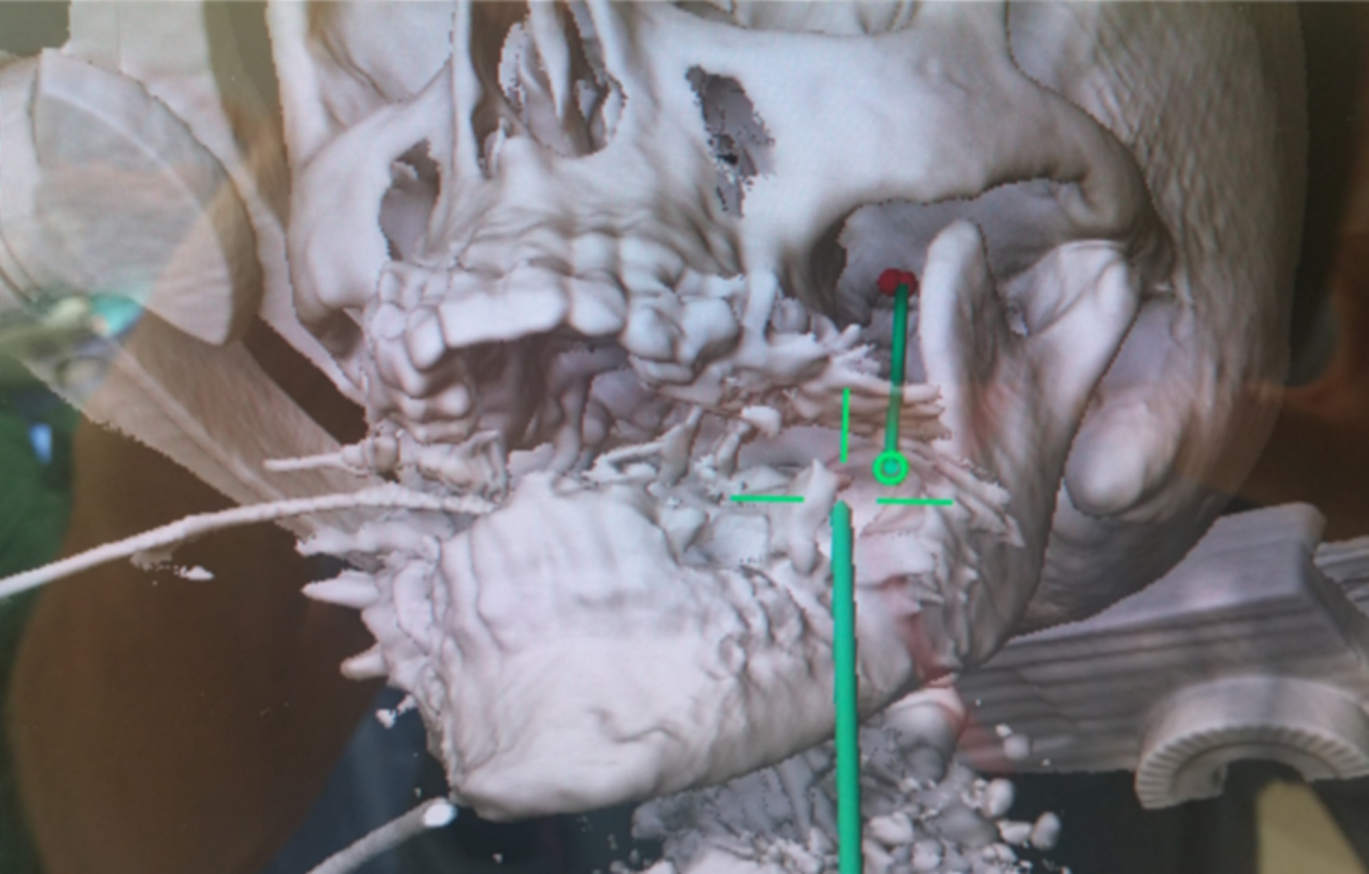
Intraoperative neuronavigation demonstrating three-dimensional reconstruction of the face and skull base, target as the foramen ovale (in red), and proposed trajectory (in green).

**Figure 3:**
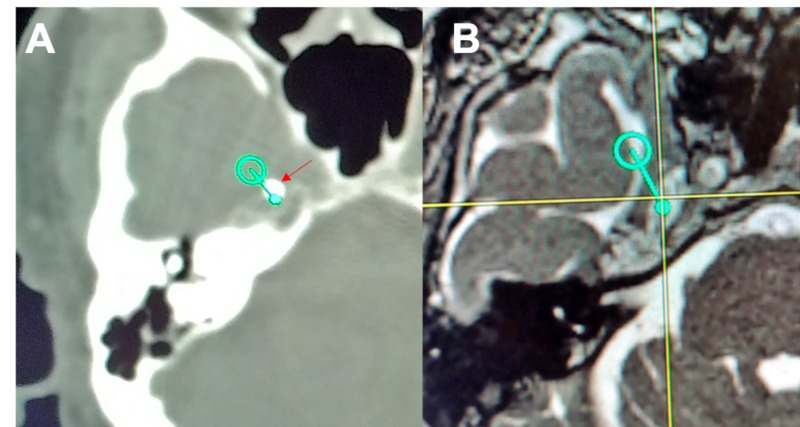
Intraoperative confirmation of adequate needle placement (A) Post-needle placement intraoperative CT showing position of needle tip (arrow) as seen on the stereotactic planning computer. The open green circle indicates the relative position of the foramen ovale (on a different slice). The thin green line represents the trajectory from the foramen ovale to the target in Meckel's cave. The solid green circle indicates the intersection of the trajectory on this CT slice. It can be seen that the needle tip is on the trajectory line between the foramen ovale and the intended target, just short of the planned target. (B) This is a derived image from the stereotactic planning computer showing a preoperative T2 MRI scan slice fused with the intraoperative CT slice just shown. The stereotactic coordinates of the needle tip are transposed as cross-hair to this MRI image of Meckel's cave, with planned trajectory shown in green. Deviation of around 2 mm between the needle tip coordinates and the target in Meckel's cave was noted. The needle tip is clearly in Meckel's cave, and there was good CSF return at this location. No further adjustments to the needle were made, and glycerol was injected with excellent patient outcome.

Percutaneous entry point is identified using anatomical landmarks defined by Hartel [9]. The Brainlab software will show a proposed trajectory (offset) passing medial to the ramus and coronoid process of the mandible and lateral to the pterygoid plate [19]. As different variants of this general technique were tried, we found improved success and efficiency when the trajectory was planned such that Meckel’s cave was set as the target and the foramen ovale was set as the entry point. The spinal needle was guided toward the foramen ovale with the assistance of neuronavigation. Figure 1 demonstrates the identification of the foramen ovale in all three planes as seen on the Brainlab stereotactic planning computer with fused high-resolution preoperative CT. Figure 2 depicts a three-dimensional trajectory on intraoperative CT reconstruction of the face and skull base. Advancement of the needle is guided by virtual visualization on neuronavigation and haptic feedback. We confirmed that the needle did not transverse the intraoral mucosa through direct visualization and palpation. The needle then "engages" once the tip is in the foramen ovale. This is inferred by neuronavigation. The tip is advanced slightly, approximately 5 mm, to target Meckel's cave, which is further along the proposed trajectory with the final position within the foramen ovale. Proper positioning is suggested by CSF flow from Meckel's cave when obtained. Another Brainlab intraoperative CT is performed to confirm the final needle position before injecting glycerol. This scan may be fused with the preoperative MRI to aid in identifying the needle tip position relative to the target in Meckel's cave, as shown in Figure 3. This "confirmatory" scan is useful in cases of false-negative and false-positive CSF flow. In the case of false-negative, there is no CSF flow despite correct needle placement through the foramen ovale. Theorectically, this may be more common in our population of patients who have undergone previous procedures and may have experienced scarring of Meckel's cave preventing spontaneous CSF flow.

After correct needle placement is confirmed, the patient is placed on a transport gurney with careful attention paid to keep the spinal needle sterile and in an exact position. The head is raised to a near vertical position so that the glycerol dependently settles in the floor of Meckel's cave. Around 0.25 to 0.65 mg of glycerol is injected through the spinal needle. The spinal needle is then removed. In our technique, glycerol injection does not require intraoperative anesthesia emergence; rather, the patient undergoes general anesthesia with endotracheal tube or LMA airway until injection is completed. The patient's head is kept in a chin down position for 15 minutes so as to let the glycerol "marinate" the trigeminal nerve and semilunar ganglion. After anesthesia emergence and airway removal, the patient is then advised to keep their head in this position for four hours post-operatively to optimize results.

Cases 

Patient demographic and cohort characteristics are presented in Tables 1 and 2. Average age of our cohort was 64.5 years, and 71% (10 of 14) were females. Of the patients, 92.3% (13 of 14) had classic TN-1 symptoms, and 85.7% (12 of 14) of patients had undergone a previous surgical treatment and 33.3% (4 of 12) of patients had undergone two or more procedures prior to glycerol rhizotomy. Patients 13 and 14 underwent glycerol rhizotomy as a first attempt due to patient preference. In 50% of patients, we were unable to obtain CSF flow intraoperatively but were able to confirm correct needle placement on CT in all cases prior to injecting glycerol. Five of these seven patients had undergone previous surgical treatment including microvascular decompression or radiosurgery or a combination of both. Interestingly, in two surgically naïve patients, CSF flow was not obtained intraoperatively. In all seven patients who demonstrated a lack of intraoperative CSF, all demonstrated improved facial pain at post-operative follow-up visit. At two-week post-operative follow-up, 92.3% (12 of 13) of patients experienced relief of facial pain. A repeat surgical procedure was eventually performed in 46% (6 of 13) of patients. Time to repeat procedure ranged from 2 to 12 months, with an average of 6 months. All cases of glycerol rhizotomy were technically successful in cannulating the foramen.

**Table 1:** Demographic data CSF, cerebrospinal fluid; F, female; RS, radiosurgery; NF, neurofibromatosis; MVD, microvascular decompression; GR, glycerol rhizotomy; M, male

Patient	Age/Sex	Comorbidities	Distribution	Previous surgery	Medical treatments	Intraoperative CSF return	Improved pain post-operative	Interval to repeat surgery
1	80/F	-	V2, V3	RS	Carbamazepine, gabapentin	Yes	Yes	-
2	53/F	-	V2, V3	RS	Carbamazepine	Yes	Yes	-
3	34/F	NF	V2, V3	MVD	Carbamazepine, gabapentin	No	Yes	10 months
4	84/F	-	V2	RS	Carbamazepine	Yes	Yes	-
5	82/F	-	V2, V3	RS	Carbamazepine	No	Yes	11 months
6	56/F	MS	V3	MVD, GR	Carbamazepine	Yes	No	2 months
7	71/F	-	V1, V2, V3	MVD, RS, GR x 2	Topiramate, gabapentin	Yes	Yes	4 months
8	51/M	-	V1, V2, V3	MVD	Carbamazepine, nortriptyline	Yes	Yes	12 months
9	66/M	-	V1, V2	RS	Gabapentin, oxcarbamazepine	Yes	Unknown	-
10	69/F	-	V1, V2, V3	MVD, RS	Carbamazepine	No	Yes	-
11	82/F	-	V1, V2	MVD	Carbamazepine, gabapentin	No	Yes	4 months
12	53/F	-	V1, V2, V3	MVD, RS x 2	Oxcarbamazepine, amitriptyline	No	Yes	-
13	59/M	-	V1, V2, V3	-	Gabapentin	No	Yes	-
14	63/M	-	V2, V3	-	Gabapentin	No	Yes	-

**Table 2:** Cohort characteristics CSF, cerebrospinal fluid

Average age (years)	64.5	
	Percentage	Proportion
Female	71.0	10/14
TN-1 class	93.0	13/14
Previous surgical therapy	85.7	12/14
Intraoperative CSF flow present	50.0	7/14
Post-surgical improvement	92.3	12/13
Underwent subsequent procedure	46.2	6/13

We will now review select cases in which there was no CSF flow noted intraoperatively but cannulation was successfully achieved and confirmed.

Patient 3

A 34-year-old female presented with a history of right V1 and V2 trigeminal neuralgia and neurofibromatosis (NF) who underwent a successful microvascular decompression five years earlier and then again in the year prior to glycerol rhizotomy with no improvement in her symptoms. Her V1 and V2 symptoms recurred a few years later. The patient did not desire another microvascular decompression, and therefore we elected to pursue glycerol rhizotomy. Her right foramen ovale was sclerotic and stenosed, which was perhaps related to her NF diagnosis. Multiple trajectories were attempted, but we experienced difficulty in engaging the foramen due to the atypical configuration. Dimensions of her right foramen ovale were 0.90 cm (major diameter) x 0.31 cm (minor diameter), whereas the left-sided dimensions were 0.94 cm (major diameter) x 0.41 cm (minor diameter). Finally, we found a more inferosuperior trajectory that landed on the lateral aspect of the foramen on imaging. CSF flow was not obtained with advancement of the needle, and we were unable to advance the needle at a certain point secondary to the short length of the needle in relation to her full cheek from this angle. Glycerol was injected at two different sites: (1) 0.25 mL at the most superior/deepest point and (2) 0.15 mL at a more inferior/shallow point nearest the foramen ovale.

Patient 5

An 82-year-old female presented with a history of left V3 trigeminal neuralgia and had previously undergone radiosurgery six months prior and had residual numbness in the left face. She underwent a trigeminal glycerol rhizotomy. Her cannulation was quite difficult. On our initial attempt, we engaged the foramen ovale but did not obtain CSF flow. A second CT scan was performed showing the needle within the foramen ovale. The needle was advanced and rotated, and slow flow of CSF was obtained. Another CT was performed and confirmed adequate positioning. Around 0.25 mL of glycerol was injected.

Patient 11 

An 82-year-old female presented with right-sided V1, V2 pain with questionable vascular compression. She did not desire to undergo craniotomy due to her age and preferred glycerol rhizotomy. Cannulation was difficult, and initial cannulation was in the middle fossa floor, just lateral to the foramen ovale. This was confirmed by a CT scan. Anecdotally, penetrating osteoporotic bone of the middle fossa floor may feel similar to engaging the foramen ovale. There was no drainage of blood or CSF from the spinal needle. The needle was then withdrawn and repositioned at a more inferior trajectory, and we were able to engage the foramen well; this was confirmed on CT prior to injecting glycerol. Upon review of the case, it was concluded that the standard trajectory was misdirected because of her being edentulous.

In two cases, the jaw anatomy presented obstacles: In patient 1, we anticipated that her severe temporomandibular joint arthritis would be a barrier to cannulation; however, we did not experience difficulties, perhaps in part due to neuronavigation. Patient 11’s trajectory was altered by edentulousness (as noted previously).

In patient 7, we were better able to target V1 and V2 within Meckel's cave with the use of intraoperative navigation and subsequent intraoperative CT.

## Discussion

Our cohort had a high pain relief rate of 92.3% at two-week follow-up. Glycerol rhizotomy offers short-term efficacy in this challenging patient population, in which 85.7% have previously undergone surgical intervention. Other similar series show a pain relief rate of 80.4% [3].

In all 14 patients, cannulation was successfully accomplished with the use of intraoperative CT guidance. In patients 1 and 11, the jaw anatomy presented technical nuances. Patient 3 proved to be our most difficult cannulation and provided four insights: (1) the aberrant anatomy of her foramen ovale may have contributed to difficulty with cannulation, (2) the trajectory to engage the foramen was modified to finally engage the foramen, (3) CSF flow is not always present despite needle placement within Meckel's cave and confirmatory imaging is helpful in these cases, and (4) mandibular anatomy is a limiting factor in this neurosurgical approach, and, in this case, cheek fullness limited our needle depth within Meckel's cave [20]. In patient 5, we encountered difficulty accessing Meckel's cave and obtaining CSF flow. We were successful on our second attempt with the aid of intraoperative navigation and repeat intraoperative imaging. In patient 7, we were better able to target V1 and V2 within Meckel's cave with the use of intraoperative navigation and subsequent intraoperative CT. Patient 7 demonstrated that more accurate localization of V1 and V2 could be achieved with our protocol. In patient 11, her edentulousness led to a misguided cannulation of the middle fossa floor. This problem was identified and the trajectory was corrected to engage the foramen. These insights are summarized in Table 3.

**Table 3:** Insights and considerations CSF, cerebrospinal fluid

Category	Insights
Oral and facial	Obesity with cheek fullness may limit the working length of the spinal needle; alternately, a longer needle is more difficult to guide Mandibular anatomy can complicate needle trajectory Edentulous patients may require a non-standard entry point to achieve an ideal trajectory
Foramen ovale	Foramen ovale may be anatomically “constrained” by sclerosis, small size or spurs resulting in difficult cannulation
Navigation	Navigation aids in understanding of the foramen ovale anatomy (shape, size, obstructive bony spurs) Trajectory can be simulated prior to cannulation and may assist in overcoming anatomical obstacles Further refinements of needle depth can be confirmed to selectively target V1, V2, and/or V3 branches
Confirmation imaging	Repeat intraoperative imaging of needle placement, especially in the case of negatory CSF flow, can be useful in confirming needle placement.

CSF flow could not be obtained in half of all cases in this series. In two cases, this occurred in surgically naïve patients demonstrating that this may be a common issue in the salvage population, but it may also occur in non-salvage patients. The use of confirmatory CT scan is useful in corroborating correct needle placement before injection. In all patients lacking intraoperative CSF flow, pain was improved at the post-operative follow-up, which further corroborates adequate cannulation.

## Conclusions

This report details the application of CT neuronavigation for glycerol trigeminal rhizotomy and experience with 14 patients. Due to availability, safety, and ease of application, we advocate the use of intraoperative CT for both neuronavigation guidance and intraoperative confirmation of foramen ovale cannulation and needle tip position in Meckel's cave.
